# Heterogeneity in Longitudinal Links Between Social Support and Cognitive Development: A Person-Centered Analysis of Chinese Early Adolescents

**DOI:** 10.3390/bs16050723

**Published:** 2026-05-08

**Authors:** Wenshan Yu, Xiaodong Zeng, Ying Ren

**Affiliations:** Faculty of Education, Beijing Normal University, Beijing 100088, China; yuwenshan@mail.bnu.edu.cn (W.Y.);

**Keywords:** social support, cognitive development, person-centered analysis, heterogeneity, latent profile analysis, Chinese early adolescents

## Abstract

Social support from parents, teachers, and peers has been linked to adolescent cognitive development, but these associations may vary across individuals. This study examined whether longitudinal links between multi-source social support and cognitive ability differ across Chinese early adolescents with distinct personality-related profiles. Using two-wave data from the China Education Panel Survey (CEPS; N = 6415 seventh graders), we conducted latent profile analysis and identified four heuristic personality-related profiles: resilient, undercontrolled, reserved, and overcontrolled. Multi-group cross-lagged panel models showed significant subgroup heterogeneity in the overall regression structure. In the undercontrolled, reserved, and overcontrolled subgroups, peer support was the only support source prospectively associated with later cognitive ability, whereas parental support showed a unique prospective association with later cognitive ability in the resilient subgroup. Positive reverse paths from cognitive ability to later support appeared only in the resilient and reserved subgroups; in the overcontrolled subgroup, a small negative path from cognitive ability to perceived teacher support was observed instead. Interconnections among parental, teacher, and peer support also varied descriptively across subgroups. Although the cross-lagged coefficients were modest, these findings suggest structured heterogeneity in longitudinal links between social support and cognitive ability that may be obscured in aggregate models.

## 1. Introduction

A central question in adolescent development research is not only whether social support contributes to cognitive development, but also whether such associations are uniform across young people. A substantial body of research has linked perceived social support to cognitive functioning, self-regulated learning, academic engagement, and achievement (Costa-Cordella et al., 2021; Martínez-López et al., 2023; Tao et al., 2022; Wu et al., 2023). At the same time, these associations are often estimated at the population level, implicitly assuming that adolescents respond to relational resources in broadly similar ways. Yet developmental heterogeneity is plausible, and approaches that relax strong homogeneity assumptions may provide a more informative account of when, for whom, and through which relational pathways support matters (Howard & Hoffman, 2018; Woo et al., 2024).

### 1.1. Social Support, Dispositional Heterogeneity, and Cognitive Ability

One reason average associations may be insufficient is that adolescents are embedded in multiple, partially overlapping relational contexts. From a bioecological perspective, developmental processes are shaped not by a single source of support in isolation but by the joint operation of family, school, and peer environments (Bronfenbrenner & Morris, 2007; Skinner et al., 2022). Recent person-centered studies likewise suggest that adolescents differ in their configurations of perceived support across sources, and that these configurations are meaningfully associated with academic and psychosocial functioning (Chan et al., 2022; Petersen et al., 2023; Hernández et al., 2025). This implies that support may matter not only because of its absolute level, but also because of how different sources combine within adolescents’ broader relational ecologies.

Prior findings are also not fully consistent across support sources. Meta-analytic evidence indicates robust average associations between teacher support and academic engagement or achievement (Tao et al., 2022). By contrast, recent longitudinal research suggests that peer support may become more relevant during early adolescence, especially for academic functioning embedded in everyday school life (Gao et al., 2025; Villar et al., 2025). In addition, person-centered studies indicate that the relative importance of family, teacher, and peer support may depend on adolescents’ broader support configurations rather than remain fixed across all individuals (Chan et al., 2022; Hernández et al., 2025). These inconsistencies suggest that the developmental relevance of specific support sources may vary across subgroups of adolescents.

A useful way to conceptualize such variation is to consider how dispositional characteristics shape adolescents’ engagement with different relational contexts. These characteristics are relevant to the present argument because they capture broad individual differences in how adolescents participate in classroom and peer interactions, experience school connectedness, regulate emotion, and sustain academic persistence. These features may shape how adolescents perceive and use available support, as well as how their competence, engagement, or need for support becomes visible to parents, teachers, and peers. Recent longitudinal evidence is consistent with this relational view of dispositional heterogeneity. Clark et al. (2023) showed reciprocal associations between personality traits and peer-group characteristics in adolescence, while Brandmiller et al. (2024) demonstrated that teachers’ perceptions of student motivation and engagement are prospectively linked to student outcomes. In the Chinese educational context, these dynamics may be amplified by several features of early secondary schooling: academic standing is often salient to students and parents, peer relationships are closely tied to achievement processes, and parental involvement is often organized around school performance (Megalokonomou & Zhang, 2024; Shen & French, 2024; Yang, 2025). Under these conditions, the same support source may not carry the same developmental relevance across adolescents. Peer support may be closely tied to everyday classroom participation and peer positioning, whereas teacher support may depend partly on whether competence and engagement become visible in instructional interactions.

These considerations are also relevant to reciprocal pathways. Supportive relationships may precede later cognitive functioning, and adolescents’ prior functioning may in turn be linked to later perceived support from parents, teachers, and peers (Brandmiller et al., 2024; T. Chen et al., 2025; Yang, 2025). Such reverse associations are unlikely to be universal. Cognitive competence may become developmentally consequential in social settings only when it is behaviorally expressed and noticed. Adolescents who differ in behavioral visibility, interpersonal style, or emotional stability may therefore show different cognition-to-support patterns even when their cognitive ability is comparable.

To examine whether such heterogeneity is systematically patterned, person-centered methods can complement variable-centered analyses when the goal is to identify subgroups characterized by similar multivariate configurations rather than to assume population homogeneity (Handley et al., 2024; Howard & Hoffman, 2018; Woo et al., 2024). In the present study, we use personality-related indicators as a subgrouping strategy because dispositional characteristics are theoretically relevant to both sides of the support–cognition link. These indicators may capture broad differences in how adolescents engage with parental, teacher, and peer support, and in how their competence becomes visible within family, classroom, and peer contexts. Empirically, personality-related configurations resembling resilient, overcontrolled, and undercontrolled patterns have been identified across adolescent samples and cultural settings (Kerber et al., 2021; So et al., 2024), and similar profile structures have been recovered in Chinese adolescent data using large-scale survey indicators comparable to those available in CEPS (H. Chen et al., 2024). Given the constraints of the survey design, the present indicators serve as broad dispositional proxies rather than as a validated personality inventory. The resulting subgroups are therefore used as data-driven analytic groupings for heterogeneity analysis, not as definitive personality types.

The present study focuses on cognitive ability rather than curriculum-based academic achievement as the focal outcome. CEPS administered both subject-specific achievement measures and a separate cognitive assessment designed to capture logical reasoning and problem-solving, scored using a three-parameter item response theory model (Pan et al., 2022). Cognitive ability is analytically preferable for the present purpose because it is less directly confounded by subject-specific instruction, teacher grading practices, and course-specific motivation than curriculum-based achievement. This does not make the measure entirely independent of schooling, but it reduces the extent to which the outcome is tied to particular instructional or evaluative processes.

This heterogeneity may be particularly important in early adolescence. The transition into middle school or lower secondary school is accompanied by changes in school organization, peer networks, parental involvement, and expectations for autonomy, making it a period in which support processes may be reorganized across contexts (Hill & Tyson, 2009; Lorijn et al., 2024; Virtanen et al., 2022). These issues are also substantively relevant in China, where school performance is closely tied to both peer relations and family involvement during early secondary schooling (Shen & French, 2024; Yang, 2025). Examining whether reciprocal associations between support and cognitive development vary across adolescent subgroups is therefore theoretically and empirically timely.

### 1.2. The Current Study

The present study addresses these gaps by combining latent profile analysis (LPA) of personality-related indicators with multi-group cross-lagged panel models (CLPMs) to examine whether the longitudinal associations among parental, teacher, and peer support and cognitive ability differ across dispositional subgroups of Chinese early adolescents. Using two-wave data from the China Education Panel Survey (CEPS), we investigate heterogeneity in both support-to-cognition and cognition-to-support associations, while also examining whether the interconnections among the three support sources vary across subgroups over time. This design is appropriate for the present aims because person-centered methods are especially useful when heterogeneity is theoretically meaningful, and because adolescent support processes unfold across multiple relational contexts rather than within a single dyad (Handley et al., 2024; Skinner et al., 2022; Woo et al., 2024).

Our study makes three contributions. First, it moves beyond average-effect models by examining whether the longitudinal links between social support and cognitive ability differ across adolescents with distinct dispositional configurations. Second, by modeling parental, teacher, and peer support within the same longitudinal framework, it captures both directional links between support and cognition and variation in the internal structure of adolescents’ support systems across subgroups. Third, by focusing on cognitive ability in a national sample of Chinese early adolescents, the study extends a literature still dominated by Western samples to a context in which school performance is highly salient for both family involvement and peer relationship processes (Shen & French, 2024; Yang, 2025).

### 1.3. Research Questions and Hypotheses

Because the subgrouping strategy is exploratory and the specific cross-lagged pathway configurations cannot be predicted precisely a priori, we combine a general heterogeneity hypothesis with more limited directional hypotheses regarding support-source salience and reciprocal dynamics.

**H1.**  *The overall pattern of longitudinal associations among parental support, teacher support, peer support, and cognitive ability differs significantly across personality-related subgroups.*

The classroom-based peer ecology motivates the following hypothesis:

**H2.**  *The prospective associations from social support to later cognitive ability differ across subgroups, and peer support is expected to show broader relevance across subgroups than teacher support during early adolescence.*

Although students’ academic position is often socially visible in Chinese junior high school classrooms, whether this visibility is reflected in later perceived support may depend on how adolescents’ dispositional characteristics shape their behavioral presentation in everyday interactions.

**H3.**  *Reverse associations from cognitive ability to later perceived support are expected to be selective rather than universal, because cognitive competence may be associated with later perceived support only when it becomes socially visible through adolescents’ behavioral presentation, interpersonal engagement, and classroom participation.*

Because the interconnections between parental, teacher, and peer support are more difficult to predict directionally within a data-driven subgrouping framework, we retain the following question as exploratory:

**RQ1.**  *Do the longitudinal interconnections among parental, teacher, and peer support differ in number and configuration of significant support-to-support paths across personality-related subgroups?*

## 2. Materials and Methods

### 2.1. Data and Participants

Data were drawn from the China Education Panel Survey (CEPS), a large-scale, nationally representative longitudinal survey of junior high school students in China administered by the National Survey Research Center at Renmin University of China. CEPS employed a stratified, multistage probability-proportional-to-size sampling design. In the baseline survey, students were sampled from 438 classrooms in 112 schools across 28 county-level units in mainland China (National Survey Research Center at Renmin University of China, n.d.). The baseline wave used in the present study was collected during the 2013–2014 academic year, and the follow-up wave during 2014–2015 (National Survey Research Center at Renmin University of China, n.d.; Yang, 2025).

The present analyses focused on the seventh-grade cohort observed across both waves. The baseline CEPS seventh-grade sample comprised 10,279 students, of whom 9449 were successfully followed at Wave 2, yielding a retention rate of 91.9% (Yang, 2025). The analytic sample was further restricted to students with complete data on the focal study variables and demographic covariates, resulting in a final sample of 6415 adolescents. Accordingly, the reduction from the original baseline cohort reflects both panel nonresponse by Wave 2 and additional complete-case restrictions for the variables used in the present analyses. Table 1 summarizes the demographic characteristics of the analytic sample overall and by latent profile.

To evaluate the potential bias introduced by the complete-case restriction, we compared the analytic sample (N = 6415) with the excluded seventh graders (N = 3864) on baseline focal variables and demographic covariates (Appendix A Table A1). Little’s test rejected the null hypothesis of Missing Completely at Random (MCAR), χ^2^ (6079) = 8513.75, *p* < 0.001. In addition, excluded students showed lower baseline cognitive ability, lower perceived parental, teacher, and peer support, lower parental education, and were more likely to be male, to hold non-local household registration, and to have siblings. These diagnostics indicate that missingness was systematic rather than completely random. The complete-case restriction limits the generalizability of the findings, because the analytic sample was systematically higher-functioning and more socioeconomically advantaged than the excluded baseline cases (Appendix A Table A1). We retained this strategy to keep the subgroup classification and multi-group CLPM analyses based on the same cases, but the results should be understood as applying primarily to students with complete data on the focal variables and covariates.

### 2.2. Measures

#### 2.2.1. Social Support

Social support was assessed using three student-reported subscales from the CEPS questionnaire, following prior research using these measures (Fang et al., 2020). Parental support was measured with four items capturing perceived emotional support from both mothers and fathers and their attentiveness to school-related issues (e.g., “How often does your mother/father discuss things that happened at school with you?”; “How often does your mother/father discuss your worries and troubles with you?”). Items were rated on a 3-point scale (1 = never to 3 = often). Cronbach’s α was 0.71 at T1 and 0.76 at T2.

Teacher support was measured with six items capturing perceived instructional attention and encouragement from subject teachers (e.g., “My mathematics teacher always asks me to answer questions in class”; “My Chinese teacher always praises me”). Items were rated on a 4-point scale (1 = strongly disagree to 4 = strongly agree). Cronbach’s α was 0.88 at T1 and 0.87 at T2.

Peer support was measured with two items capturing perceived classroom climate and peer friendliness (“Most of my classmates are nice to me”; “My class is in good atmosphere”). Items were rated on a 4-point scale (1 = strongly disagree to 4 = strongly agree). Cronbach’s α was 0.62 at T1 and 0.66 at T2. The relatively low reliability of this subscale is noted as a limitation, and the corresponding path estimates should be interpreted with caution. For all three subscales, item scores were standardized and averaged to form composite scores at each wave.

#### 2.2.2. Cognitive Ability

Cognitive ability was measured using the standardized CEPS cognitive test administered at both waves. The test was designed to assess logical reasoning and problem-solving rather than curriculum-specific knowledge, and its development and scoring procedures are documented in the CEPS technical materials. CEPS estimated students’ scores using a three-parameter item response theory model that incorporates item difficulty, discrimination, and guessing parameters, and the resulting standardized score was used as the cognitive ability indicator in all analyses (Pan et al., 2022).

#### 2.2.3. Personality-Related Indicators

To construct personality-related indicators compatible with the CEPS item pool, we organized available T1 items into five broad dispositional domains. This operational strategy is broadly consistent with recent CEPS-based research that has used the survey’s Big-Five-like items in profile-oriented analyses (H. Chen et al., 2024; Sun & Xue, 2025; Ye et al., 2024). The items were organized into five composite indicators: extraversion (1 item: “I often take part in school/class activities”), agreeableness (1 item: “I feel close to people in this school”), emotional stability (5 items assessing the absence of depressive symptoms over the past week, reverse-coded; e.g., “In the past seven days, I have felt blue”), openness (4 items assessing intellectual curiosity and self-perceived cognitive abilities; e.g., “I was a fast learner”; “I was curious about new stuff”), and conscientiousness (3 items assessing academic persistence; e.g., “I would try my best to finish even the homework I dislike”). Item scores were standardized and averaged within each dimension. Because extraversion and agreeableness were each represented by a single item, these indicators may capture only one behavioral expression of multidimensional traits rather than the full construct domain. The resulting LPA profiles may therefore partly reflect item-specific response patterns; accordingly, the profile labels should be interpreted as summaries of the observed indicator configurations rather than as descriptions of broad personality constructs. The LPA was conducted using T1 indicators only; T2 personality-related items were not used in the subgroup classification.

#### 2.2.4. Control Variables

The analyses adjusted for gender (0 = male, 1 = female), household registration location (hukou; 0 = non-local registration, 1 = local registration), only-child status (0 = non-only child, 1 = only child), and family socioeconomic status (SES). SES was constructed using principal component analysis of three indicators: household economic conditions (self-reported as poor, middle, or rich), father’s education level, and mother’s education level. The first principal component was retained as the SES composite.

### 2.3. Data Analysis

The analysis proceeded in three stages. In Stage 1, data preparation including panel matching and variable construction was conducted in Stata 17. Item scores were standardized (z-scored) and averaged within each construct to create composite indicators for the three social support dimensions and the five personality-related dimensions.

In Stage 2, latent profile analysis (LPA) was conducted in R 4.4 using the tidyLPA package. The five T1 personality-related composite indicators served as the profile inputs. We estimated solutions with one to five profiles and evaluated them using the Akaike information criterion (AIC), Bayesian information criterion (BIC), sample-size adjusted BIC, entropy, and the bootstrap likelihood ratio test. Following current methodological guidance, profile retention was based on convergence across statistical fit, classification quality, subgroup size, and substantive interpretability rather than on any single index alone (Spurk et al., 2020; Van Lissa et al., 2024). In particular, BIC and adjusted BIC were treated as especially informative for class enumeration, whereas entropy was used as an indicator of classification precision rather than as a stand-alone decision rule (Van Lissa et al., 2024). Profile labels were assigned only after final model selection and are intended as heuristic summaries of the observed indicator configurations.

**Figure 1:**
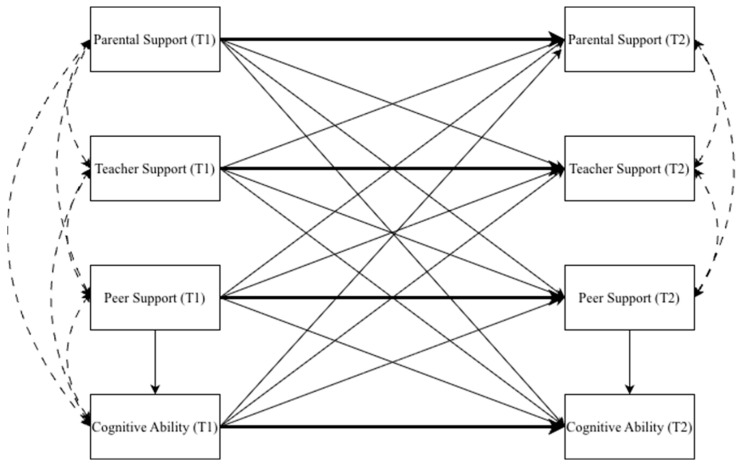
Conceptual diagram of the multi-group cross-lagged panel model. Note: Thick solid arrows represent autoregressive paths; thin solid arrows represent cross-lagged paths; dashed double-headed curves represent covariances at T1 and residual covariances at T2 (estimated only among the three support variables). Control variables (gender, hukou, and socioeconomic status) predicted selected T2 outcomes in the pruned model but are omitted from the figure for visual clarity (see Section 2.3 for details). The model was estimated separately for each of the four personality-related subgroups.

In Stage 3, multi-group cross-lagged panel models (CLPMs) were estimated in R using the lavaan package with robust maximum likelihood (MLR) estimation. The model included four observed variables at each wave: parental support, teacher support, peer support, and cognitive ability. All T1 variables were specified as correlated exogenous variables, and each T2 variable was regressed on all four T1 variables, yielding both autoregressive and cross-lagged paths within each subgroup. To assess potential multicollinearity among the Time 1 support predictors, we computed variance inflation factors (VIFs). All VIF values were below 1.16 in the full sample and below 1.11 within each subgroup (Appendix A Table A2), indicating that multicollinearity was negligible. Residual covariances among the three T2 support variables were estimated to account for shared within-wave variance not explained by the lagged predictors. Residual covariances between T2 cognitive ability and the T2 support variables were not retained in the final model in order to preserve parsimony after preliminary checks indicated minimal contribution to model fit. The structure of the final multi-group CLPM is illustrated in Figure 1.

Because the publicly available CEPS data used in the present study provided only two panel waves for the focal variables, we estimated a traditional cross-lagged panel model rather than a random-intercept cross-lagged panel model. A traditional CLPM can be estimated with two-wave panel data and is useful for examining prospective longitudinal associations conditional on prior levels of the variables (Orth et al., 2021). Accordingly, the present coefficients are interpreted as subgroup-differentiated longitudinal associations at the observed-variable level. Because the multi-group models relied on observed composites rather than latent factors, formal latent-variable tests of measurement invariance were not implemented within the present analytic framework. Unmodeled measurement errors may also have affected both cross-lagged and autoregressive estimates. The unequal reliabilities of the support subscales (α = 0.62–0.88) further suggest that measurement precision differed across support sources.

Model pruning: We first estimated a full model in which all four demographic covariates (gender, hukou, only-child status, and SES) predicted all four T2 outcomes within each subgroup. To improve parsimony, the final model retained only those covariate paths that were significant across preliminary specifications and substantively relevant to the focal outcomes. Specifically, SES and hukou were retained as predictors of T2 parental support; gender was retained as a predictor of T2 peer support; and SES, hukou, and gender were retained as predictors of T2 cognitive ability. This pruning reduced the number of estimated parameters from 240 to 200 while preserving the core cross-lagged pattern. As a robustness check, the full-covariate model yielded substantively identical cross-lagged results (see Appendix A Table A3 for comparison).

Heterogeneity test: To assess whether the structural paths differed across subgroups, we compared an unconstrained model (regression paths freely estimated across groups) with a constrained model (paths set equal across groups) using a scaled chi-square difference test appropriate for MLR estimation (Rosseel, 2012). The omnibus test evaluates all path constraints simultaneously; therefore, we interpret the omnibus comparison in conjunction with subgroup-specific path estimates and significance patterns.

## 3. Results

### 3.1. Descriptive Statistics and Correlations

Table 2 presents descriptive statistics and bivariate correlations for all study variables. Parental support, teacher support, peer support, and cognitive ability were generally positively correlated within and across waves. Cross-wave correlations of the same construct were moderate, including parental support (r = 0.489), teacher support (r = 0.433), peer support (r = 0.370), and cognitive ability (r = 0.512), indicating substantial temporal stability while leaving room for cross-lagged associations. Among the three support sources, teacher support showed the weakest correlations with cognitive ability, whereas peer support showed the strongest concurrent correlations with cognitive ability at both waves.

### 3.2. Latent Profile Analysis

**Figure 2:**
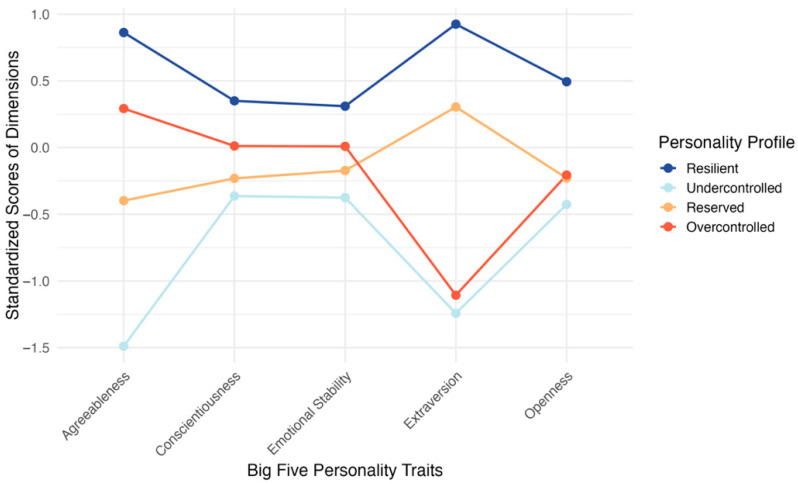
Standardized Personality-Related Indicator Profiles Across the Four Subgroups. **Note**: The figure displays standardized scores on the five personality-related indicators used in the latent profile analysis. Subgroup labels are heuristic descriptors of relative dispositional configurations and are not intended as definitive personality categories.

Table 3 summarizes the latent profile analysis results. The four-profile solution was selected as the preferred solution because it showed the lowest AIC and BIC values, high entropy (0.997), and a significant bootstrap likelihood ratio test. By contrast, the five-profile solution showed poorer fit and a nonsignificant bootstrap likelihood ratio test. The four subgroups were also of adequate size for subsequent multi-group analyses, ranging from 16.68% to 35.62% of the sample. Figure 2 displays the standardized personality-related indicator patterns across the four subgroups. The very high entropy value (0.997) indicates near-certain classification, reducing concern that classification uncertainty materially affected the downstream multi-group analyses, although it does not address the construct validity of the profile indicators.

Following the relative configuration of the five indicators, the four subgroups were labeled as resilient, undercontrolled, reserved, and overcontrolled. The resilient subgroup (*n* = 2285; 35.62%) showed the highest scores across all five indicators. The undercontrolled subgroup (*n* = 1070; 16.68%) showed the lowest scores overall, especially on agreeableness and emotional stability. The reserved subgroup (*n* = 1844; 28.75%) showed generally moderate scores across indicators. The overcontrolled subgroup (*n* = 1216; 18.96%) showed relatively low extraversion and openness, alongside moderate agreeableness and conscientiousness. These labels are used as heuristic descriptors of relative dispositional configurations rather than as definitive personality categories.

### 3.3. Multi-Group Cross-Lagged Panel Models

The final pruned multi-group cross-lagged panel model showed the following fit indices: χ^2^ (72) = 558.07, *p* < 0.001; CFI = 0.932; TLI = 0.803; RMSEA = 0.065 (90% CI [0.060, 0.070]); SRMR = 0.040. Taken together, these indices indicate mixed fit: CFI, RMSEA, and SRMR were within commonly accepted ranges, whereas TLI remained below conventional thresholds. Recent methodological work cautions against mechanically applying fixed fit-index cutoffs across models, because indices such as CFI, TLI, RMSEA, and SRMR are sensitive to model characteristics and data conditions, including complexity, degrees of freedom, and indicator structure (Goretzko et al., 2024; Groskurth et al., 2024). We interpret the model as providing a usable but not unproblematic basis for careful comparison of subgroup-specific path patterns. The lower TLI nevertheless indicates remaining model misfit and therefore supports our cautious emphasis on broad path patterns rather than fine-grained comparisons of individual coefficients. Relative to the fuller specification, the pruned model reduced the number of estimated parameters from 240 to 200 while preserving the core cross-lagged pattern.

To assess overall subgroup heterogeneity, we compared the unconstrained model with a model in which regression paths were constrained to equality across subgroups. The omnibus comparison was significant, Δχ^2^ (66) = 91.02, *p* = 0.022, indicating that the regression structure was not fully equivalent across the four subgroups and providing formal support for H1. However, this omnibus test constrains all regression paths simultaneously and does not imply that every path differs across groups. Inspection of the subgroup-specific estimates suggests that the observed heterogeneity was concentrated in a limited set of theoretically relevant paths, especially the support-to-cognition and cognition-to-support associations. As a robustness check, the core cross-lagged pattern remained substantively similar in the fuller model.

Before turning to the subgroup-specific path estimates, a brief note on effect magnitudes is warranted. Across the four subgroups, the significant cross-lagged coefficients were modest in size, ranging from β = −0.058 to β = 0.090. Their practical significance should not be overstated, particularly in a large sample that provides substantial power to detect relatively small prospective associations. That said, the substantive contribution of the present analysis lies less in the magnitude of any single coefficient than in the patterned heterogeneity in the subgroup-specific relevance of different support sources.

Across all four subgroups, autoregressive paths were consistently larger than the cross-lagged coefficients, indicating substantial temporal stability alongside more modest prospective associations across constructs. Table 4 presents the standardized estimates from the final pruned model, with emphasis on the support-to-cognition and cognition-to-support paths that contributed most clearly to the observed subgroup heterogeneity. Full unstandardized and standardized estimates are reported in Appendix A Table A4.

#### 3.3.1. Social Support Predicting Cognitive Ability

The longitudinal associations from social support to later cognitive ability differ across the four subgroups. In the resilient subgroup, parental support was the only support source prospectively associated with subsequent cognitive ability (β = 0.065, *p* = 0.001). In contrast, in the undercontrolled (β = 0.090, *p* < 0.001), reserved (β = 0.058, *p* = 0.006), and overcontrolled (β = 0.084, *p* = 0.003) subgroups, peer support emerged as the only significant support source prospectively associated with later cognitive ability. Teacher support did not significantly predict later cognitive ability in any subgroup. Taken together, these findings suggest a subgroup-differentiated pattern of modest prospective associations: parental support reached statistical significance only in the resilient subgroup, whereas the peer-support path was significant in the other three subgroups.

#### 3.3.2. Cognitive Ability Predicting Social Support

The reverse associations from cognitive ability to later support also varied across subgroups. In the resilient subgroup, cognitive ability positively predicted later parental support (β = 0.041, *p* = 0.037). In the reserved subgroup, cognitive ability positively predicted both later teacher support (β = 0.043, *p* = 0.042) and later peer support (β = 0.050, *p* = 0.025). No significant reverse paths were observed in the undercontrolled subgroup. In the overcontrolled subgroup, cognitive ability negatively predicted later teacher support (β = −0.058, *p* = 0.026). Notably, this was the only negative cross-lagged path in the entire model. Overall, these results indicate that cognition-to-support associations were more selective and subgroup-specific than support-to-cognition associations.

#### 3.3.3. Interconnections Among Support Sources

As an exploratory descriptive analysis, we examined whether the longitudinal links among parental, teacher, and peer support varied across subgroups (see Appendix A Table A5 for the full set of estimates with confidence intervals). Across most subgroups, parental and teacher support formed the most consistent core of the support network, with the parental-to-teacher path reaching significance in all subgroups. Beyond this common pattern, the number of significant support-to-support paths differed across groups. The reserved subgroup showed six significant directional paths, the resilient subgroup five, the undercontrolled subgroup four, and the overcontrolled subgroup three. This ordering should be interpreted cautiously, as it is based on counting significant individual paths rather than on a formal statistical comparison of system-level connectivity across groups. The pattern is therefore presented as a descriptive observation about variation in support-system interconnectedness rather than as a formally tested group difference.

#### 3.3.4. Autoregressive Stability

All autoregressive paths were significant across profiles. Parental support showed the strongest stability among the support sources, with standardized coefficients ranging from 0.402 to 0.474. Cognitive ability also showed strong temporal stability, with coefficients ranging from 0.450 to 0.525. Teacher support showed moderate stability (0.295 to 0.388), and peer support showed the lowest but still significant stability (0.204 to 0.303).

## 4. Discussion

This study examined whether longitudinal associations among parental support, teacher support, peer support, and cognitive ability differed across personality-related subgroups of Chinese early adolescents. The omnibus chi-square difference test for the final model was significant, indicating that the cross-lagged structure was not fully equivalent across the four subgroups. The subgroup-specific patterns were concentrated in three areas: which support source was linked to subsequent cognitive ability, whether cognitive ability was linked to later support, and how the three support sources were interconnected over time. Because the study relied on a two-wave observed-variable CLPM, these findings are interpreted as subgroup-differentiated longitudinal associations rather than as evidence of within-person or causal processes.

### 4.1. Peer Support and Later Cognitive Ability

Peer support showed subgroup-specific relevance for later cognitive ability. Specifically, in the undercontrolled, reserved, and overcontrolled subgroups, peer support at T1 was the only support source with a statistically significant prospective link to T2 cognitive ability. This pattern is consistent with recent work showing that peer support and peer relationship quality are associated with adolescents’ academic engagement, motivational regulation, and achievement-related functioning (Gao et al., 2025; Villar et al., 2025). It is also in line with person-centered evidence indicating that support from school friends may differentiate adolescents’ academic performance in early adolescence (Hwang et al., 2024). Taken together, this pattern is consistent with H2, which anticipated that peer support would show broader relevance across subgroups than teacher support during early adolescence. At the same time, these cross-lagged estimates were small in magnitude, and the peer-support measure captured perceived classroom climate and peer friendliness more broadly than individualized peer support. Given the two-item scale and its modest reliability, this pattern is best interpreted as suggestive evidence of peer-related subgroup differences, not as a definitive estimate of peer-support effects.

The resilient subgroup was the exception to this pattern. For these adolescents, parental support was the only support source with a statistically significant prospective link to later cognitive ability, and this association was bidirectional. One possible interpretation is that adolescents with more adaptive dispositional configurations may be better positioned to engage with structured parental support, and their cognitive competence may in turn be associated with sustained parental involvement. This reading is in line with longitudinal evidence on reciprocal links between parental support processes and academic functioning among Chinese adolescents (T. Chen et al., 2025; Xu, 2025), as well as person-oriented work showing that more supportive parenting environments are associated with more favorable developmental trajectories (Teuber et al., 2022). The contrast with the other three subgroups suggests that the relative importance of parental versus peer support may differ across adolescents in ways that are obscured in aggregate models.

### 4.2. Cognitive Ability and Perceived Teacher Support

One finding that warrants cautious interpretation was the negative path from T1 cognitive ability to T2 perceived teacher support in the overcontrolled subgroup. This was the only negative cross-lagged coefficient in the model. Adolescents in this subgroup were characterized by relatively low extraversion and openness alongside moderate agreeableness, a configuration that may be less behaviorally salient in everyday classroom interaction than more outwardly expressive profiles. One possible reading is that cognitively capable students whose competence is expressed in less visible ways may perceive less overt teacher attention or support over time. This possibility is broadly compatible with longitudinal evidence showing that teacher perceptions of students’ motivation and engagement are linked to later student outcomes, and that such perceptions are shaped by characteristics beyond achievement alone (Brandmiller et al., 2024). Given the small effect, the fact that this path appeared in only one subgroup, and the absence of direct measures of teacher behavior or classroom interaction, this result is best regarded as a tentative, hypothesis-generating observation rather than evidence for a specific explanatory mechanism.

### 4.3. Support-Network Connectivity Across Subgroups

Subgroup heterogeneity also appeared in the broader interconnections among parental, teacher, and peer support over time, not only in the focal support-to-cognition and cognition-to-support paths. In descriptive terms, the reserved subgroup showed the largest number of significant support-to-support paths, followed by the resilient, undercontrolled, and overcontrolled subgroups. This ordering requires caution. It is based on the number of statistically significant paths rather than on a formal statistical comparison of system-level coherence across groups.

Nevertheless, the results suggest that subgroup heterogeneity may extend beyond the focal links between social support and cognitive ability to the broader configuration of adolescents’ support ecologies. Recent person-centered work indicates that combinations of support sources, rather than the level of any single source considered in isolation, may differentiate adolescents’ academic and psychological functioning (Chan et al., 2022; Hwang et al., 2024). However, the current data do not include direct measures of cross-context coordination, fit, or relational reciprocity. The support system differences reported here are better viewed as descriptive structural contrasts that warrant further investigation, rather than as evidence that particular subgroups possess more tightly integrated or more mutually reinforcing support systems.

### 4.4. Selectivity of Cognition-to-Support Associations and Subgrouping Approach

Taken together, the subgroup-specific reverse associations from cognitive ability to later support were selective rather than universal. Positive cognition-to-support paths appeared only in the resilient subgroup and the reserved subgroup, whereas no such paths emerged in the undercontrolled subgroup and a negative path was observed in the overcontrolled subgroup. This selectivity is partly consistent with H3 and reinforces the value of examining heterogeneity rather than assuming that prior cognitive functioning is associated with later perceived support in similar ways across all adolescents.

Nevertheless, the subgrouping variables were derived from survey-based personality-related indicators rather than a standardized personality inventory. The four subgroups are therefore best viewed as broad, data-driven analytic groupings used to reveal heterogeneity, not as validated personality types. The reserved subgroup, in particular, does not map neatly onto the canonical resilient–overcontrolled–undercontrolled taxonomy and thus warrants replication. Even so, the subgrouping strategy still revealed patterned heterogeneity in which support sources were associated with later cognition, cognition was associated with later support, and how support-system linkages differed across groups.

### 4.5. Implications for Theory and Educational Practice

Theoretically, the present findings suggest that personality-related subgrouping may serve as a useful analytic lens for examining heterogeneity in the longitudinal links between social support and cognitive functioning. The value of the person-centered approach lies in showing how dispositional configurations are linked to adolescents’ positions within relational ecologies, beyond differences in mean levels of dispositional indicators. This interpretation is consistent with bioecological and person-centered perspectives, which emphasize that development is shaped by the joint operation of family, school, and peer contexts rather than by any single source of support in isolation (Skinner et al., 2022; Chan et al., 2022). In the Chinese early secondary context, academic standing is salient to students and parents, and peer relationships are closely tied to achievement processes. The repeated association between peer support and later cognitive ability in several subgroups may reflect a broader classroom ecology in which academic standing, peer positioning, and perceived support are closely connected (Megalokonomou & Zhang, 2024; Shen & French, 2024). The subgroup-specific cognition-to-support paths are compatible with the possibility that cognitive ability is not uniformly associated with later perceived support across adolescents, although this interpretation remains tentative given the observed-variable design and modest effect sizes.

Beyond these theoretical considerations, the results raise several practical questions that merit cautious attention. They suggest that educators and school counselors should avoid assuming that the same source of support is equally relevant for all adolescents. Perceived peer support may deserve attention not only as a socioemotional resource but also as a component of the broader classroom context relevant to students’ cognitive functioning. This implication is consistent with recent evidence linking peer support to academic engagement, motivational regulation, and achievement-related functioning among adolescents (Gao et al., 2025; Villar et al., 2025). The negative cognition-to-teacher-support association observed in the overcontrolled subgroup further suggests a practical issue worth monitoring: academically capable but less behaviorally visible students may not always perceive sustained teacher support in the same way as more outwardly engaged peers. Rather than implying a direct intervention prescription, this finding points to the value of teacher awareness, classroom observation, and support practices that attend not only to low-achieving or disruptive students but also to quieter students whose needs may be less visible.

### 4.6. Limitations

This study has several limitations. First, the analysis relied on a two-wave observed-variable cross-lagged panel model. Although this design is useful for examining prospective associations conditional on prior levels, it does not separate within-person from between-person variance. The findings should therefore be interpreted as subgroup-differentiated longitudinal associations rather than as strong causal effects. Future research using three or more waves could apply RI-CLPM or related models to examine whether similar subgroup-specific patterns emerge at the within-person level (Hamaker et al., 2015; Orth et al., 2021). Second, the measures imposed important constraints. The personality-related indicators were limited in scope, with extraversion and agreeableness each represented by a single item, peer support showed relatively low reliability, and the use of observed composite scores meant that measurement error was not modeled directly. This means that the subgroup classification, although statistically sharp (entropy = 0.997), may not fully correspond to the intended personality dimensions, and the replicability of the specific profile configurations with richer multi-item personality measures remains to be tested. In addition, because the analyses were conducted on observed composites, cross-group comparability of these measures was assumed rather than formally tested through latent-variable measurement invariance procedures. These features may have attenuated or destabilized some cross-lagged estimates, with the degree of bias potentially varying across support sources because their reliabilities differed. The comparative pattern across parental, teacher, and peer support should therefore be understood as a pattern of observed associations, not as precise evidence of differences in true predictive strength.

The social support and personality-related indicators were also derived from student self-reports. Although cognitive ability was measured using a standardized test, common-rater processes among the self-reported constructs may still have contributed to some of the observed associations, particularly among the support-related variables. Shared method variance therefore cannot be ruled out as a partial alternative explanation. In particular, the support-to-support cross-lagged paths and the concurrent correlations among the three support sources may partly reflect common-rater variance in addition to substantive associations. The support-to-cognition and cognition-to-support paths are less directly susceptible to this specific concern because cognitive ability was measured using a standardized test rather than student self-report. Future studies should examine these questions with richer multi-item measures and latent-variable designs. Third, the analytic sample was derived through complete-case analysis. Supplemental diagnostics indicated that missingness was not completely at random, and compared with excluded baseline cases, the analytic sample appeared somewhat higher-functioning and more socioeconomically advantaged. This pattern suggests possible selection bias and somewhat narrower generalizability to lower-functioning segments of the baseline cohort. Future work could address this issue more directly through designs and estimation strategies that make fuller use of partially observed data. Finally, although the core cross-lagged pattern was stable across the full and pruned models, model fit was not uniformly strong, particularly with respect to TLI, and the data were collected from Chinese seventh graders in 2013–2015. The results should therefore be generalized cautiously across historical periods, age groups, and educational contexts. It will be important to test whether comparable patterns can be observed in more recent cohorts and in other educational settings.

## 5. Conclusions

The present study shows that the longitudinal associations between social support and cognitive development are not uniform across adolescents. Using a person-centered subgrouping strategy, we found that the relative relevance of parental, teacher, and peer support, the selectivity of cognition-to-support associations, and the pattern of support-system interconnections varied across personality-related subgroups. These findings suggest that average-effect models may obscure structured heterogeneity in how support and cognitive development are linked over time. Although the cross-lagged coefficients were modest and the two-wave observed-variable design does not permit strong causal inference, the study contributes to the literature by documenting personality-related subgrouping as a potentially informative, data-driven lens for examining heterogeneity in support–cognition associations. Practically, the findings suggest that educational support should not assume a uniform relevance of parental, teacher, and peer support across adolescents. Future research using stronger longitudinal and measurement designs should examine whether these subgroup-specific patterns can be replicated and whether they translate into practically meaningful differences in support needs.

## Figures and Tables

**Table 1:** Demographic Characteristics of the Analytic Sample (N = 6415).

Characteristic	Full Sample	Resilient (*n* = 2285)	Undercontrolled (*n* = 1070)	Reserved (*n* = 1844)	Overcontrolled (*n* = 1216)
Gender					
Female	3195 (49.8%)	1217 (53.3%)	474 (44.3%)	932 (50.5%)	572 (47.0%)
Male	3220 (50.2%)	1068 (46.7%)	596 (55.7%)	912 (49.5%)	644 (53.0%)
Hukou					
Local registration	5228 (81.5%)	1874 (82.0%)	873 (81.6%)	1487 (80.6%)	994 (81.7%)
Non-local registration	1187 (18.5%)	411 (18.0%)	197 (18.4%)	357 (19.4%)	222 (18.3%)
Only child					
Yes	3054 (47.6%)	1233 (54.0%)	405 (37.9%)	895 (48.5%)	521 (42.8%)
No	3361 (52.4%)	1052 (46.0%)	665 (62.1%)	949 (51.5%)	695 (57.2%)
Family SES (M ± SD)	0.00 ± 1.35	−0.25 ± 1.37	0.33 ± 1.20	−0.02 ± 1.39	0.20 ± 1.25

Note. SES = socioeconomic status, constructed via principal component analysis. Hukou reflects whether the student’s household registration is in the local county/district.

**Table 2:** Descriptive Statistics and Bivariate Correlation for Study Variables.

Variables	1	2	3	4	5	6	7	8
1. Parent Support T1	-							
2. Parent Support T2	0.489 ***	-						
3. Teacher Support T1	0.269 ***	0.211 ***	-					
4. Teacher Support T2	0.225 ***	0.293 ***	0.433 ***	-				
5. Peer Support T1	0.219 ***	0.203 ***	0.306 ***	0.224 ***	-			
6. Peer Support T2	0.166 ***	0.201 ***	0.197 ***	0.356 ***	0.370 ***	-		
7. Cognitive Ability T1	0.125 ***	0.118 ***	0.066 ***	0.061 ***	0.151 ***	0.082 ***	-	
8. Cognitive Ability T2	0.136 ***	0.154 ***	0.051 *	0.036	0.178 ***	0.107 ***	0.512 ***	-
M	2.09	2.11	2.68	2.48	3.27	3.23	0.13	0.43
SD	0.53	0.53	0.70	0.72	0.70	0.70	0.85	0.76

Note: T1 = wave1; T2 = wave2. * *p* < 0.05. *** *p* < 0.001.

**Table 3:** Fit Indices for Latent Profile Analysis Models.

Number of Profiles	LogLik	AIC	BIC	SABIC	Entropy	BLRT *p*
1	−45,510.0	-	-	-	-	-
2	−43,923.0	87,878.0	87,986.0	87,936.0	0.639	0.0099
3	−42,224.0	84,491.0	84,640.0	84,570.0	0.999	0.0099
4	−41,179.0	82,414.0	82,604.0	82,515.0	0.997	0.0099
5	−41,798.0	83,663.0	83,893.0	83,785.0	0.780	0.9800

Note: AIC = Akaike Information Criterion; BIC = Bayesian Information Criterion; SABIC = Sample-Size Adjusted BIC; BLRT = Bootstrap Likelihood Ratio Test. The four-profile solution was retained for subsequent analyses based on fit, entropy, interpretability, and subgroup size.

**Table 4:** Standardized Path Estimates from the Final Pruned Multi-Group Cross-Lagged Panel Model.

Predictor (Time 1) → Outcome (Time 2)	Resilient	Undercontrolled	Reserved	Overcontrolled
Support → Cognitive Ability				
Parental Support → Cognitive Ability	0.065 ** [0.026, 0.103]	0.030 [−0.021, 0.081]	0.042 [−0.000, 0.084]	0.030 [−0.018, 0.079]
Teacher Support → Cognitive Ability	−0.028 [−0.065, 0.009]	−0.030 [−0.084, 0.023]	−0.036 [−0.079, 0.008]	−0.048 [−0.101, 0.006]
Peer Support → Cognitive Ability	0.025 [−0.019, 0.069]	0.090 *** [0.041, 0.140]	0.058 ** [0.016, 0.100]	0.084 ** [0.028, 0.139]
Cognitive Ability → Support				
Cognitive Ability → Parental Support	0.041 * [0.003, 0.079]	0.039 [−0.017, 0.094]	0.032 [−0.009, 0.073]	0.000 [−0.050, 0.049]
Cognitive Ability → Teacher Support	0.026 [−0.012, 0.065]	−0.029 [−0.087, 0.029]	0.043 * [0.002, 0.085]	−0.058 * [−0.109, −0.007]
Cognitive Ability → Peer Support	0.016 [−0.026, 0.058]	−0.020 [−0.079, 0.040]	0.050 * [0.006, 0.094]	−0.005 [−0.061, 0.050]
Autoregressive Paths (Stability)				
Parental Support → Parental Support	0.431 *** [0.395, 0.468]	0.474 *** [0.427, 0.522]	0.437 *** [0.397, 0.478]	0.402 *** [0.349, 0.454]
Teacher Support → Teacher Support	0.344 *** [0.302, 0.386]	0.327 *** [0.267, 0.386]	0.388 *** [0.344, 0.433]	0.295 *** [0.239, 0.351]
Peer Support → Peer Support	0.248 *** [0.198, 0.298]	0.204 *** [0.140, 0.267]	0.303 *** [0.255, 0.350]	0.247 *** [0.190, 0.305]
Cognitive Ability → Cognitive Ability	0.461 *** [0.426, 0.497]	0.525 *** [0.478, 0.573]	0.450 *** [0.411, 0.490]	0.478 *** [0.430, 0.526]

Note: Entries are standardized regression coefficients (β) with 95% confidence intervals. Only the focal cross-lagged paths linking social support and cognitive ability, together with autoregressive paths, are shown in the main table. Support-to-support paths are reported in Appendix A Table A5. * *p* < 0.05. ** *p* < 0.01. *** *p* < 0.001.

## Data Availability

The data presented in this study can be accessed through the China National Social Survey Data Archive on 1 November 2024 (https://www.cnsda.org/index.php) by searching for the “China Education Panel Survey.” The project numbers for the two waves of data are [72810330] and [61662993]. Further information about the China Education Panel Survey project is available at http://ceps.ruc.edu.cn/.

## Appendix A

**Table A1:** Comparison of the Analytic Sample and Excluded Baseline Cases.

Variable	Analytic Sample	Excluded Cases	Test Statistic	*p*	Effect Size
Cognitive ability T1	0.134 (0.851)	−0.222 (0.863)	t = 20.36	<0.001	d = 0.415
Parental support T1	2.091 (0.535)	1.965 (0.566)	t = 11.12	<0.001	d = 0.229
Teacher support T1	2.681 (0.702)	2.592 (0.758)	t = 5.90	<0.001	d = 0.122
Peer support T1	3.271 (0.704)	3.072 (0.795)	t = 12.68	<0.001	d = 0.264
Mother’s education	4.061 (2.010)	3.576 (1.871)	t = 12.34	<0.001	d = 0.250
Father’s education	4.432 (2.024)	3.921 (1.896)	t = 12.86	<0.001	d = 0.260
Gender (% male)	50.2%	57.0%	χ^2^ = 42.82	<0.001	V = 0.065
Hukou (% local)	81.5%	76.9%	χ^2^ = 29.95	<0.001	V = 0.055
Only child (% yes)	47.6%	35.3%	χ^2^ = 149.17	<0.001	V = 0.120

Note: Continuous variables are presented as M (SD). Excluded cases refer to baseline seventh graders who were not included in the analytic sample because of panel nonresponse and/or missing data on one or more focal variables or covariates. Effect sizes are reported as Cohen’s d for mean comparisons and Cramér’s V for categorical comparisons.

**Table A2:** Variance Inflation Factors (VIFs) for the Time 1 Predictors.

Panel A. Full-Sample VIFs				
**T1 Predictor**	**VIF (3 Support)**		**VIF (3 Support + Cognitive)**	
Parental support	1.102		1.113	
Teacher support	1.158		1.158	
Peer support	1.129		1.146	
Cognitive ability	—		1.034	
Panel B. Subgroup-Specific VIFs for the Three Support Predictors				
**Subgroup**	***n***	**VIF Parental**	**VIF Teacher**	**VIF Peer**
Resilient	2285	1.044	1.073	1.052
Undercontrolled	1070	1.040	1.039	1.003
Reserved	1844	1.046	1.045	1.035
Overcontrolled	1216	1.051	1.101	1.053

Note: VIF = variance inflation factor. Panel A reports full-sample VIFs for the Time 1 predictors entered simultaneously in the cross-lagged regressions. Panel B reports subgroup-specific VIFs for the three Time 1 support predictors. All values were well below conventional thresholds, indicating negligible multicollinearity.

**Table A3:** Comparison of the Full-Covariate and Final Pruned Multi-Group CLPMs: Fit Indices and Core Standardized Path Estimates.

Fit Indices/Path Estimates	Full-Covariate Specification	Final Pruned Specification
Model Fit Indices		
χ^2^	562.78 ***	558.07 ***
df	64	72
CFI	0.93	0.932
TLI	0.738	0.803
RMSEA	0.07	0.065
SRMR	0.041	0.04
Core Cross-Lagged Paths (β)		
Profile 1: Resilient		
Parental Support (T1) → Cognitive Ability (T2)	0.064 **	0.065 **
Cognitive Ability (T1) → Parental Support (T2)	0.039 *	0.041 *
Profile 2: Undercontrolled		
Peer Support (T1) → Cognitive Ability (T2)	0.090 ***	0.090 ***
Profile 3: Reserved		
Peer Support (T1) → Cognitive Ability (T2)	0.058 **	0.058 **
Cognitive Ability (T1) → Peer Support (T2)	0.047 *	0.050 *
Cognitive Ability (T1) → Teacher Support (T2)	0.039 ^	0.043 *
Profile 4: Overcontrolled		
Peer Support (T1) → Cognitive Ability (T2)	0.081 **	0.084 **
Cognitive Ability (T1) → Teacher Support (T2)	−0.065 *	−0.058 *

Note: Standardized coefficients (β) are reported. The full-covariate specification included all covariate paths and T2 residual covariances. The pruned model trimmed non-significant covariate paths and T2 residual covariances that did not materially improve model fit to improve model parsimony. ^ *p* < 0.10, * *p* < 0.05, ** *p* < 0.01, *** *p* < 0.001.

**Table A4:** Full Unstandardized and Standardized Estimates for the Final Pruned Multi-Group CLPM.

Group	Outcome (T2)	Predictor (T1)	B [95% CI]	SE	β [95% CI]	*p*
Resilient	Parental Support	Parental Support	0.434 [0.395, 0.473]	0.020	0.431 [0.395, 0.468]	<0.001
Resilient	Parental Support	Teacher Support	0.056 [0.016, 0.096]	0.020	0.053 [0.015, 0.092]	=0.006
Resilient	Parental Support	Peer Support	0.068 [0.010, 0.126]	0.030	0.044 [0.006, 0.081]	=0.022
Resilient	Parental Support	Cognitive Ability	0.048 [0.003, 0.092]	0.023	0.041 [0.003, 0.079]	=0.037
Resilient	Parental Support	SES	−0.046 [−0.073, −0.020]	0.013	−0.065 [−0.103, −0.028]	<0.001
Resilient	Parental Support	Hukou	0.050 [−0.042, 0.143]	0.047	0.020 [−0.017, 0.056]	=0.283
Resilient	Teacher Support	Parental Support	0.073 [0.033, 0.114]	0.021	0.072 [0.032, 0.112]	<0.001
Resilient	Teacher Support	Teacher Support	0.362 [0.316, 0.407]	0.023	0.344 [0.302, 0.386]	<0.001
Resilient	Teacher Support	Peer Support	0.090 [0.026, 0.154]	0.033	0.057 [0.017, 0.098]	=0.006
Resilient	Teacher Support	Cognitive Ability	0.031 [−0.015, 0.077]	0.023	0.026 [−0.012, 0.065]	=0.184
Resilient	Peer Support	Parental Support	0.047 [0.010, 0.085]	0.019	0.053 [0.011, 0.094]	=0.013
Resilient	Peer Support	Teacher Support	0.016 [−0.023, 0.054]	0.020	0.017 [−0.025, 0.059]	=0.432
Resilient	Peer Support	Peer Support	0.343 [0.273, 0.412]	0.035	0.248 [0.198, 0.298]	<0.001
Resilient	Peer Support	Cognitive Ability	0.017 [−0.027, 0.060]	0.022	0.016 [−0.026, 0.058]	=0.453
Resilient	Peer Support	Gender	0.116 [0.049, 0.182]	0.034	0.067 [0.029, 0.105]	<0.001
Resilient	Cognitive Ability	Parental Support	0.047 [0.019, 0.076]	0.014	0.065 [0.026, 0.103]	=0.001
Resilient	Cognitive Ability	Teacher Support	−0.021 [−0.050, 0.007]	0.014	−0.028 [−0.065, 0.009]	=0.138
Resilient	Cognitive Ability	Peer Support	0.028 [−0.022, 0.078]	0.025	0.025 [−0.019, 0.069]	=0.271
Resilient	Cognitive Ability	Cognitive Ability	0.392 [0.356, 0.428]	0.018	0.461 [0.426, 0.497]	<0.001
Resilient	Cognitive Ability	SES	−0.063 [−0.081, −0.045]	0.009	−0.122 [−0.158, −0.086]	<0.001
Resilient	Cognitive Ability	Hukou	−0.040 [−0.105, 0.026]	0.033	−0.022 [−0.057, 0.014]	=0.233
Resilient	Cognitive Ability	Gender	0.030 [−0.021, 0.081]	0.026	0.021 [−0.015, 0.057]	=0.253
Undercontrolled	Parental Support	Parental Support	0.473 [0.420, 0.525]	0.027	0.474 [0.427, 0.522]	<0.001
Undercontrolled	Parental Support	Teacher Support	0.062 [0.006, 0.117]	0.028	0.061 [0.006, 0.116]	=0.029
Undercontrolled	Parental Support	Peer Support	0.060 [0.015, 0.106]	0.023	0.069 [0.017, 0.121]	=0.009
Undercontrolled	Parental Support	Cognitive Ability	0.044 [−0.019, 0.107]	0.032	0.039 [−0.017, 0.094]	=0.170
Undercontrolled	Parental Support	SES	−0.034 [−0.078, 0.010]	0.022	−0.042 [−0.095, 0.012]	=0.127
Undercontrolled	Parental Support	Hukou	−0.005 [−0.132, 0.122]	0.065	−0.002 [−0.052, 0.048]	=0.942
Undercontrolled	Teacher Support	Parental Support	0.137 [0.077, 0.196]	0.030	0.136 [0.078, 0.194]	<0.001
Undercontrolled	Teacher Support	Teacher Support	0.332 [0.271, 0.394]	0.031	0.327 [0.267, 0.386]	<0.001
Undercontrolled	Teacher Support	Peer Support	−0.001 [−0.055, 0.053]	0.027	−0.001 [−0.062, 0.060]	=0.970
Undercontrolled	Teacher Support	Cognitive Ability	−0.034 [−0.100, 0.033]	0.034	−0.029 [−0.087, 0.029]	=0.320
Undercontrolled	Peer Support	Parental Support	0.028 [−0.040, 0.096]	0.035	0.025 [−0.035, 0.085]	=0.415
Undercontrolled	Peer Support	Teacher Support	0.117 [0.044, 0.190]	0.037	0.103 [0.038, 0.167]	=0.002
Undercontrolled	Peer Support	Peer Support	0.200 [0.136, 0.265]	0.033	0.204 [0.140, 0.267]	<0.001
Undercontrolled	Peer Support	Cognitive Ability	−0.025 [−0.102, 0.051]	0.039	−0.020 [−0.079, 0.040]	=0.518
Undercontrolled	Peer Support	Gender	0.076 [−0.048, 0.200]	0.063	0.034 [−0.021, 0.090]	=0.227
Undercontrolled	Cognitive Ability	Parental Support	0.024 [−0.017, 0.066]	0.021	0.030 [−0.021, 0.081]	=0.250
Undercontrolled	Cognitive Ability	Teacher Support	−0.025 [−0.069, 0.019]	0.023	−0.030 [−0.084, 0.023]	=0.272
Undercontrolled	Cognitive Ability	Peer Support	0.064 [0.029, 0.100]	0.018	0.090 [0.041, 0.140]	<0.001
Undercontrolled	Cognitive Ability	Cognitive Ability	0.486 [0.431, 0.540]	0.028	0.525 [0.478, 0.573]	<0.001
Undercontrolled	Cognitive Ability	SES	−0.050 [−0.086, −0.015]	0.018	−0.075 [−0.129, −0.022]	=0.005
Undercontrolled	Cognitive Ability	Hukou	−0.068 [−0.176, 0.040]	0.055	−0.033 [−0.085, 0.019]	=0.217
Undercontrolled	Cognitive Ability	Gender	0.006 [−0.075, 0.086]	0.041	0.003 [−0.046, 0.053]	=0.891
Reserved	Parental Support	Parental Support	0.441 [0.398, 0.484]	0.022	0.437 [0.397, 0.478]	<0.001
Reserved	Parental Support	Teacher Support	0.058 [0.013, 0.103]	0.023	0.054 [0.013, 0.096]	=0.011
Reserved	Parental Support	Peer Support	0.103 [0.054, 0.151]	0.025	0.089 [0.047, 0.130]	<0.001
Reserved	Parental Support	Cognitive Ability	0.037 [−0.011, 0.084]	0.024	0.032 [−0.009, 0.073]	=0.128
Reserved	Parental Support	SES	−0.061 [−0.088, −0.033]	0.014	−0.088 [−0.127, −0.048]	<0.001
Reserved	Parental Support	Hukou	0.160 [0.065, 0.254]	0.048	0.066 [0.027, 0.104]	<0.001
Reserved	Teacher Support	Parental Support	0.083 [0.038, 0.127]	0.023	0.082 [0.038, 0.126]	<0.001
Reserved	Teacher Support	Teacher Support	0.416 [0.365, 0.467]	0.026	0.388 [0.344, 0.433]	<0.001
Reserved	Teacher Support	Peer Support	0.086 [0.034, 0.138]	0.027	0.074 [0.029, 0.119]	=0.001
Reserved	Teacher Support	Cognitive Ability	0.050 [0.002, 0.099]	0.025	0.043 [0.002, 0.085]	=0.042
Reserved	Peer Support	Parental Support	0.070 [0.026, 0.114]	0.022	0.068 [0.025, 0.112]	=0.002
Reserved	Peer Support	Teacher Support	0.090 [0.036, 0.143]	0.027	0.083 [0.034, 0.133]	=0.001
Reserved	Peer Support	Peer Support	0.353 [0.296, 0.410]	0.029	0.303 [0.255, 0.350]	<0.001
Reserved	Peer Support	Cognitive Ability	0.058 [0.007, 0.110]	0.026	0.050 [0.006, 0.094]	=0.025
Reserved	Peer Support	Gender	0.138 [0.059, 0.216]	0.040	0.071 [0.031, 0.111]	<0.001
Reserved	Cognitive Ability	Parental Support	0.034 [−0.000, 0.068]	0.017	0.042 [−0.000, 0.084]	=0.053
Reserved	Cognitive Ability	Teacher Support	−0.030 [−0.068, 0.007]	0.019	−0.036 [−0.079, 0.008]	=0.112
Reserved	Cognitive Ability	Peer Support	0.054 [0.015, 0.092]	0.020	0.058 [0.016, 0.100]	=0.006
Reserved	Cognitive Ability	Cognitive Ability	0.417 [0.373, 0.460]	0.022	0.450 [0.411, 0.490]	<0.001
Reserved	Cognitive Ability	SES	−0.083 [−0.105, −0.061]	0.011	−0.149 [−0.189, −0.110]	<0.001
Reserved	Cognitive Ability	Hukou	−0.076 [−0.153, 0.002]	0.040	−0.039 [−0.079, 0.001]	=0.056
Reserved	Cognitive Ability	Gender	−0.004 [−0.066, 0.057]	0.031	−0.003 [−0.043, 0.037]	=0.892
Overcontrolled	Parental Support	Parental Support	0.409 [0.354, 0.464]	0.028	0.402 [0.349, 0.454]	<0.001
Overcontrolled	Parental Support	Teacher Support	0.063 [−0.000, 0.126]	0.032	0.057 [−0.000, 0.114]	=0.051
Overcontrolled	Parental Support	Peer Support	0.051 [−0.013, 0.115]	0.033	0.043 [−0.011, 0.098]	=0.121
Overcontrolled	Parental Support	Cognitive Ability	−0.000 [−0.058, 0.057]	0.029	−0.000 [−0.050, 0.049]	=0.989
Overcontrolled	Parental Support	SES	−0.075 [−0.115, −0.035]	0.020	−0.096 [−0.146, −0.045]	<0.001
Overcontrolled	Parental Support	Hukou	0.050 [−0.074, 0.174]	0.063	0.020 [−0.029, 0.068]	=0.428
Overcontrolled	Teacher Support	Parental Support	0.130 [0.078, 0.181]	0.026	0.137 [0.083, 0.191]	<0.001
Overcontrolled	Teacher Support	Teacher Support	0.303 [0.244, 0.362]	0.030	0.295 [0.239, 0.351]	<0.001
Overcontrolled	Teacher Support	Peer Support	0.082 [0.022, 0.142]	0.031	0.075 [0.020, 0.130]	=0.008
Overcontrolled	Teacher Support	Cognitive Ability	−0.063 [−0.119, −0.008]	0.028	−0.058 [−0.109, −0.007]	=0.026
Overcontrolled	Peer Support	Parental Support	0.090 [0.033, 0.146]	0.029	0.091 [0.034, 0.148]	=0.002
Overcontrolled	Peer Support	Teacher Support	0.020 [−0.044, 0.084]	0.032	0.019 [−0.041, 0.078]	=0.537
Overcontrolled	Peer Support	Peer Support	0.281 [0.213, 0.349]	0.035	0.247 [0.190, 0.305]	<0.001
Overcontrolled	Peer Support	Cognitive Ability	−0.006 [−0.068, 0.056]	0.032	−0.005 [−0.061, 0.050]	=0.849
Overcontrolled	Peer Support	Gender	0.119 [0.019, 0.218]	0.051	0.062 [0.010, 0.114]	=0.019
Overcontrolled	Cognitive Ability	Parental Support	0.023 [−0.014, 0.059]	0.019	0.030 [−0.018, 0.079]	=0.219
Overcontrolled	Cognitive Ability	Teacher Support	−0.039 [−0.082, 0.005]	0.022	−0.048 [−0.101, 0.006]	=0.080
Overcontrolled	Cognitive Ability	Peer Support	0.072 [0.025, 0.120]	0.024	0.084 [0.028, 0.139]	=0.003
Overcontrolled	Cognitive Ability	Cognitive Ability	0.410 [0.360, 0.459]	0.025	0.478 [0.430, 0.526]	<0.001
Overcontrolled	Cognitive Ability	SES	−0.059 [−0.088, −0.030]	0.015	−0.102 [−0.153, −0.051]	<0.001
Overcontrolled	Cognitive Ability	Hukou	−0.019 [−0.109, 0.072]	0.046	−0.010 [−0.058, 0.038]	=0.684
Overcontrolled	Cognitive Ability	Gender	−0.004 [−0.074, 0.066]	0.036	−0.003 [−0.051, 0.045]	=0.902

Note: This appendix table reports both unstandardized coefficients (B) and standardized coefficients (β) with 95% confidence intervals for all regression paths in the final pruned multi-group CLPM. B estimates are unstandardized coefficients based on the modeled variable scales used in the final pruned CLPM. Social support variables were modeled as standardized composite scores, and cognitive ability was modeled using the standardized CEPS IRT-based score. Confidence intervals are 95% intervals. *p* values are two-tailed.

**Table A5:** Standardized Support-to-Support Cross-Lagged Paths in the Final Pruned Multi-Group CLPM.

Predictor (T1) → Outcome (T2)	Resilient	Undercontrolled	Reserved	Overcontrolled
Teacher Support → Parental Support	0.053 ** [0.015, 0.092]	0.061 * [0.006, 0.116]	0.054 * [0.013, 0.096]	0.057 [−0.000, 0.114]
Peer Support → Parental Support	0.044 * [0.006, 0.081]	0.069 ** [0.017, 0.121]	0.089 *** [0.047, 0.130]	0.043 [−0.011, 0.098]
Parental Support → Teacher Support	0.072 *** [0.032, 0.112]	0.136 *** [0.078, 0.194]	0.082 *** [0.038, 0.126]	0.137 *** [0.083, 0.191]
Peer Support → Teacher Support	0.057 ** [0.017, 0.098]	−0.001 [−0.062, 0.060]	0.074 ** [0.029, 0.119]	0.075 ** [0.020, 0.130]
Parental Support → Peer Support	0.053 * [0.011, 0.094]	0.025 [−0.035, 0.085]	0.068 ** [0.025, 0.112]	0.091 ** [0.034, 0.148]
Teacher Support → Peer Support	0.017 [−0.025, 0.059]	0.103 ** [0.038, 0.167]	0.083 ** [0.034, 0.133]	0.019 [−0.041, 0.078]

Note: Entries are standardized coefficients (β) with 95% confidence intervals from the final pruned multi-group CLPM. * *p* < 0.05, ** *p* < 0.01, *** *p* < 0.001.
